# An adversarial training framework for mitigating algorithmic biases in clinical machine learning

**DOI:** 10.1038/s41746-023-00805-y

**Published:** 2023-03-29

**Authors:** Jenny Yang, Andrew A. S. Soltan, David W. Eyre, Yang Yang, David A. Clifton

**Affiliations:** 1 Institute of Biomedical Engineering, Department of Engineering Science, University of Oxford, Oxford, England; 2 John Radcliffe Hospital, Oxford University Hospitals NHS Foundation Trust, Oxford, England; 3 RDM Division of Cardiovascular Medicine, University of Oxford, Oxford, England; 4 Big Data Institute, Nuffield Department of Population Health, University of Oxford, Oxford, England; 5 School of Public Health, Shanghai Jiao Tong University School of Medicine, Shanghai, China; 6 Oxford-Suzhou Centre for Advanced Research (OSCAR), Suzhou, China

**Keywords:** Medical ethics, Public health

## Abstract

Machine learning is becoming increasingly prominent in healthcare. Although its benefits are clear, growing attention is being given to how these tools may exacerbate existing biases and disparities. In this study, we introduce an adversarial training framework that is capable of mitigating biases that may have been acquired through data collection. We demonstrate this proposed framework on the real-world task of rapidly predicting COVID-19, and focus on mitigating site-specific (hospital) and demographic (ethnicity) biases. Using the statistical definition of equalized odds, we show that adversarial training improves outcome fairness, while still achieving clinically-effective screening performances (negative predictive values >0.98). We compare our method to previous benchmarks, and perform prospective and external validation across four independent hospital cohorts. Our method can be generalized to any outcomes, models, and definitions of fairness.

## Introduction

A fundamental observation in machine learning (ML) research is that models can become biased based on the samples used during training. This can lead to poorer predictive performance and unfair decision-making. Here, we define “bias” as a difference in performance between subgroups for a predictive task^1,2^; and similarly, define an “unfair” decision as any result that is skewed towards a particular group or population^2–4^. In other words, given a classifier which predicts labels *y*_*i*_ from features *x*_*i*_ for samples *i*, bias arises when a statistical property for the distribution of {*y*_*i*_, *i ϵ Z*} differs from the distribution of {*y*_*i*_, *i ϵ Z’*}, where *Z* is considered a sensitive subgroup (i.e., a group that a model may be biased against) and *Z’* is its non-sensitive complement. With respect to fairness, previous machine learning works have evaluated statistical properties such as demographic parity, equality of odds, and equal opportunity^2–6^.

If a machine learning model acquires unintentional biases, it may be unable to capture the true relationship between the features and the target outcome. This is particularly harmful in sensitive domains such as healthcare because: (1) a biased model can lead to inaccurate predictions for critical and, potentially, life-altering decisions; (2) a bias against a particular group can result in those patients receiving poorer care compared to those in other groups; and (3) a biased model can exacerbate and propagate existing inequities in healthcare and society. Thus, in our study, we propose a framework for bias mitigation using adversarial debiasing, whereby a model is trained to learn parameters that do not infer sensitive features. We consider a classifier which predicts *y*_*i*_ from features *x*_*i*_, while remaining unbiased with respect to some sensitive feature, *Z*. To evaluate group outcome fairness, we use the statistical metric of equality of odds, which states that a classifier *Ŷ* is fair if *Ŷ* and *Z* are conditionally independent given *Y*^2–5^. For binary classification, this is equivalent to P(*Ŷ* = 1|*Y* = *y*, *Z* = 0) = P(*Ŷ* = 1|*Y* = *y*, *Z* = 1), *y ϵ* {0, 1}. Using the real-world clinical task of COVID-19 screening, we demonstrate the effectiveness of this technique for two sensitive features - patient ethnicity and hospital location.

Previous works on training fair machine learning systems have shown that ML models can be trained to reduce demographic-based biases. Such biases are highly relevant in clinical settings, as they can unintentionally arise through admission bias, sampling bias, or observer bias, which can collectively result in data that is unrepresentative of the general population^7,8^. For example, in terms of gender bias, physicians have been found to have an unconscious bias for ascribing the symptoms of coronary heart disease (CHD) among women to some other disorder^9^; and when the same proportion of women and men presented with chest pain, an observational study found that women were 2.5 times less likely to be referred to a cardiologist for management^10^. Similarly, it was shown that physicians tended to ask fewer diagnostic questions and prescribe the fewest CHD-related medications to middle-aged women^11^. In terms of ethnic bias, a systematic review of USA-based studies found that in the emergency room, black patients were 40% less likely to receive pain medication than white patients^12^. When such biases are present in training data (and subsequently, learned during training), models have been found to perform unequally across different patient populations^13^, and even negatively impact those in underrepresented groups^14^. For example, if a model was designed to determine who to prescribe CHD-related medications, men might be selected to receive the majority of them, further deepening inequities in healthcare. In addition to poorer health and treatment outcomes, this issue is also relevant with respect to privacy preservation and statistical disclosure, as some regions may have a very small number of patients of a given ethnicity; and thus, if a machine learning model is biased against this group, there is an increased probability of identifying these patients.

Another feature on which models can become biased on is the location where samples have been collected. Specifically, with respect to healthcare, studies have found that clinical outcomes and practice can vary across geographic regions and between hospitals. Namely, disease prevalence/mortality, quality of healthcare services, and specific devices used (such as brands of blood analysis devices) can vary widely across hospitals in different regions. This heterogeneity has been acknowledged worldwide and has been examined for a range of medical conditions and diseases^15–17^, as well as different drivers of healthcare quality^15,18^. Thus, a model trained using data from one hospital may not generalise well to data at a different hospital, as the methods used to collect, process, and organize the respective datasets may have unintentionally encoded site-specific biases (this is typically referred to as measurement bias)^2,19^. For example, it has previously been demonstrated that state-of-the-art machine learning methods consistently underdiagnosed under-served patient populations^1^. To address such biases, many ML projects may integrate datasets from multiple sources in order to increase the amount of data available for training (as reaching generalizability typically requires a large training set). Recently, researchers have shown that federated learning (FL) could effectively predict clinical outcomes of COVID-19, using combined data from multiple sites^20^. However, with respect to both the curation of datasets and FL, different centres may have varying amounts of training data available, resulting in a skewed dataset on which site-specific biases may be accumulated. And specifically, in the case of FL, site-specific biases can still be present in a final model since it aggregates the weights of each independently-trained (i.e., site-specific) model. If such biases become reflected in a model’s decisions, then certain hospitals could be unintentionally isolated for exhibiting poorer outcomes, further widening interregional and interhospital inequality gaps.

There have been significant advancements in the area of machine learning fairness and bias mitigation, with techniques typically fitting into one of three categories: pre-processing, in-processing, and post-processing. Pre-processing methods perform bias mitigation directly on the training data itself (before ever reaching an ML model); in-processing methods perform bias mitigation during the model training process; and post-processing methods perform bias mitigation on trained models^21^.

Pre-processing bias mitigation methods include sampling^22,23^ and perturbation methods^24,25^. Sampling methods can either increase the chances of overfitting since it utilizes exact copies of the minority class (leading to solutions that tend towards memorization rather than learning how to differentiate between classes); or wastes potentially useful information through undersampling the minority class^26^. Moreover, sampling changes the true prevalence and distribution, which can affect the outcome and any subsequent calibration. Similarly, perturbation methods which adjust the values of different groups (in attempt to bring them closer together), changes the underlying distribution of the data. Although this may resolve some disparity between groups, it can be viewed as synthetically modifying the data; thus, making it harder to translate into practice, as there are not always measures and evaluation metrics for the clinical quality of synthetic data^27^.

Post-processing bias mitigation methods are applied after a model has already been successfully trained. These include creating new models (after seeing a biased one) with modified features or weights^28–30^ and relabelling predictions to satisfy fairness constraints^31–33^. Although these methods can improve on a biased model, creating and retraining new or modified models can be slow and expensive (which can be overwhelming for hospitals), especially for tasks requiring very large computational power; and relabelling does not change the biased model itself, nor ensure that the outcome for the predictive task is correct. This is particularly important for clinical tasks, as a model should satisfy both fairness and predictive accuracy.

Thus, for the purposes of our study, we specifically focus on an in-processing paradigm, whereby bias mitigation is performed during the training of a model. Common in-processing methods include using regularization/constraints on a model’s loss function^34–36^, training compositional models^37–40^, and using adversarial learning^5,41,42^.

Although regularization/imposing constraints can help penalize bias, the loss function (and subsequently, the model) can become skewed towards the majority class present in the batch, due to aggregation of the errors (standard classification models which use gradient descent estimate marginal distribution with a differentiable loss function). Rather than focusing on the loss function, compositional models, instead, train independent models for each population group; however, this method is slow and expensive, and can be overwhelming for hospitals to reasonably implement. In another manner, adversarial learning (also known as adversarial debiasing), simultaneously trains a classifier and an adversary model in parallel, where the classifier is trained to predict the task at hand, and the adversary is trained to exploit a bias. When trained against one another, one can develop a fair model that is simultaneously a strong classifier. This method is also advantageous with respect to computational cost, as it does not require multiple processing steps or training multiple iterations of a model. Thus, we propose an adversarial training framework to train models that are unbiased towards sensitive features.

We demonstrate our framework on two sensitive features – hospitals in different geographic regions and patients of different ethnicities, and focus on this problem in the context of rapid COVID-19 diagnosis. Although we focus on a clinical task, the method described can be applied to many other domains where machine learning models are used to support decision-making. We compare our method to the benchmarks set by XGBoost-based models^43,44^ and RL-based models^26^, and evaluate the generalizability of our models by performing prospective and external validation across emergency admissions to four independent United Kingdom (UK) National Health Service (NHS) Trusts.

Adversarial debiasing has previously been shown to be successful in reducing gender (male versus female) bias in salary prediction^5,41^ and ethnicity (black vs white) bias in recidivism prediction^42^. There is currently no published research on the utility of adversarial debiasing in a clinical context. Additionally, all published adversarial debiasing research, thus far, has focused exclusively on debiasing binary attributes. However, in many real-world applications, it is often necessary to preserve a higher degree of granularity, as binning may not be biologically accurate and is heavily biased on the sample population. Therefore, through our study, we hope to encourage and demonstrate the effectiveness of adversarial debiasing on a wider range of prediction tasks and demographic features. To summarize, our main contributions in this paper are as follows:

- We propose a neural network-based framework based on adversarial debiasing that is capable of effectively determining COVID-19 status, while mitigating biases.
- We introduce an improved loss function to help with model convergence in the correct direction.
- We demonstrate adversarial debiasing in a clinical context, and evaluate its effectiveness across two different bias mitigation tasks - debiasing patient ethnicity and hospital location.
- We evaluate fairness on multiclass sensitive features and propose an evaluation metric to accommodate this, as most previous works have focused exclusively on binary features.
- We compare our results with related previous works, and perform external and prospective validation across four independent UK NHS hospital trusts, demonstrating the generalisability of our method.

## Results

We trained neural network models to predict the COVID-19 status for patients attending hospital emergency departments (ED). Previous works investigated machine learning-based methods for rapidly identifying patients with COVID-19 using a combination of blood tests, blood gas testing, and vital signs^19,26,43,44^. The studies found that ML-based methods could rapidly detect COVID-19 amongst patients presenting to ED, and performed effectively as tests-of-exclusion (quick identification of patients who are most likely to test negative) during external validation across three NHS trusts. We aimed to build upon these existing works, developing the models with adversarial methods to effectively accomplish the same task, with the added capability of mitigating biases.

### Debiasing ethnicity

For consistency, we trained our models using the same cohorts as those used in^26,44^. Accordingly, for the training and validation sets used in the ethnicity debiasing model, we used patient presentations exclusively from Oxford University Hospitals NHS Foundation Trust (OUH). From OUH, we had two data extracts - one from the first wave of the COVID-19 epidemic in the UK (December 1, 2019 to June 30, 2020), and one from the second wave (October 1, 2020–March 6, 2021) (Supplementary Fig. 1). Due to incomplete penetrance of testing during the first wave, and imperfect sensitivity of the polymerase chain reaction (PCR) test, there is uncertainty in the viral status of patients presenting who were untested or tested negative. Thus, consistent with^26,43,44^, we matched every positive COVID-19 presentation in the training set to a set of negative controls based on age, using a ratio of 20 controls:1 positive presentation. This created a simulated disease prevalence of 5%, aligning with real COVID-19 prevalences at all four sites during the dates of data extraction (range across sites between 4.27–12.2%). Sensitivity analysis to account for uncertainty in negative PCR results improved apparent accuracy^43^.

Thus, we trained and optimized our model using 114,957 COVID-free patient presentations from OUH prior to the global COVID-19 outbreak, and 701 patient presentations during the first wave of the COVID-19 epidemic in the UK that had a positive PCR test for COVID-19. This ensured that the label of COVID-19 status was correct during training. We then validated the model on 72,223 admitted patients (4600 COVID-19 positive with confirmatory testing) across four validation cohorts (OUH “wave 2”, University Hospitals Birmingham NHS Trust [UHB], Bedfordshire Hospitals NHS Foundations Trust [BH], and Portsmouth Hospitals University NHS Trust [PUH]). A summary of each respective cohort is in Table 1.

**Table 1:** Summary population characteristics for OUH training cohorts (OUH pre-pandemic and “wave one”), prospective validation cohort (OUH), independent validation cohorts of patients admitted to three independent NHS Trusts (PUH, UHB, BH).

	OUH (pre-pandemic & “wave one” cases, to 30/06/2020)		OUH	PUH	UHB	BH
Cohort	Pre-pandemic cohort	COVID-19-cases cohort	01/10/2020-06/03/2021	01/03/2020-28/02/2021	01/12/2019-29/10/2020	01/01/2021-31/03/2021
*n*, patients	114,957	701	22,857	37,896	10,293	1177
*n*, COVID positive	0	701	2012 (8.80%)	2005 (5.29%)	439 (4.27%)	144 (12.2%)
Sex:						
- Male (%)	53,370 (46.43)	376 (53.64)	11,409 (49.91)	20839 (54.99)	4831 (46.93)	627 (53.27)
- Female (%)	61,587 (53.57)	325 (46.36)	11,448 (50.09)	17,054 (45.0)	5462 (53.07)	549 (46.64)
Age, yr (IQR)	60 (38–76)	72 (55–82)	67 (49–80)	69 (48–2)	63 (42–79)	68.0 (48–82)
Ethnicity:						
-White (%)	93,921 (81.7)	480 (68.47)	17,387 (76.07)	28,704 (75.74)	6848 (66.53)	1024 (87.0)
-Not Stated (%)	13,602 (11.83)	128 (18.26)	4127 (18.06)	8389 (22.14)	1061 (10.31)	≤10
-South Asian (%)	2754 (2.4)	22 (3.14)	441 (1.93)	170 (0.45)	1357 (13.18)	71 (6.03)
-Chinese (%)	284 (0.25)	*	51 (0.22)	42 (0.11)	41 (0.4)	≤10
-Black (%)	1418 (1.23)	25 (3.57)	279 (1.22)	187 (0.49)	484 (4.7)	36 (3.06)
-Other (%)	1840 (1.6)	34 (4.85)*	410 (1.79)	269 (0.71)	333 (3.24)	29 (2.46)
-Mixed (%)	1138 (0.99)	12 (1.71)	162 (0.71)	135 (0.36)	169 (1.64)	13 (1.1)

*indicates merging for statistical disclosure control.

From Table 1, we can see that ethnicity is heavily skewed in our training dataset, making it a possible source of bias. Although “Unknown”, “Other”, and “Mixed” are ambiguous, we kept them in both our training and validation datasets, as they constituted a high number of total COVID-19 positive cases.

**Fig. 1:**
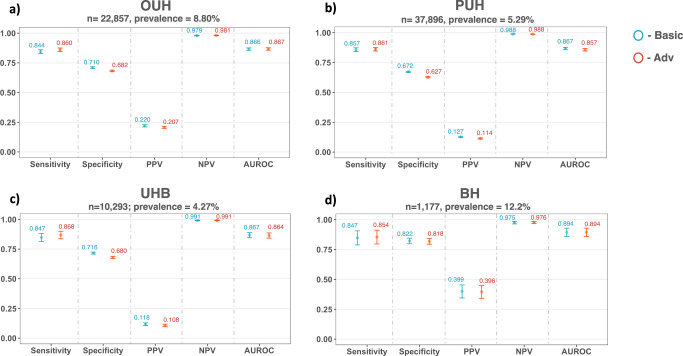
COVID-19 diagnosis performance results across different UK NHS Trusts. Panels compare performance of basic (standalone) and adversarial models (blue and red, respectively) during prospective validation (**a** OUH) and external validation (**b** PUH, **c** UHB, **d** BH). Adversarial models were trained to mitigate ethnicity biases. All models were optimized during training to achieve sensitivities of 0.9. Error bars show 95% confidence intervals. Numerical results are shown in Supplementary Table 3.

After training models on patient cohorts from OUH, we prospectively and externally validated our models across four held-out patient cohorts from OUH, PUH, UHB, and BH (results shown in Fig. 1). Using an optimized sensitivity configuration of 0.9, AUROC scores for predicting COVID-19 status were consistent across both basic and adversarial models for each cohort, achieving the highest overall performance on the BH cohort (OUH: AUROC range 0.866–0.867 [CI range 0.855–0.877]; PUH: 0.857–0.867 [0.846–0.877]; UHB: 0.864–0.867 [0.842–0.888]; BH: 0.894 [0.859–0.929]). These are comparable to the previous benchmarks reported, which used similar patient cohorts and features^26,44^ (Supplementary Table 5), demonstrating that we trained strong classifiers to begin with.

The optimized threshold also resulted in consistent scores for sensitivity across all models and cohorts (OUH: sensitivity range 0.844–0.860 [CI range 0.828–0.875]; PUH: 0.857–0.861 [0.842–0.876]; UHB: 0.847–0.868 [0.836–0.900]; BH: 0.847–0.854 [0.789–0.912]). However, there was a larger specificity range across test sites, with BH achieving the highest specificity (OUH: specificity range 0.682–0.710 [CI range 0.676–0.717]; PUH: 0.627–0.672 [0.622–0.677]; UHB: 0.680–0.716 [0.671-0.725]; BH: 0.818-0.822 [0.795-0.845]). Furthermore, as demonstrated in previous studies, our models also achieved high prevalence-dependent NPV scores (>0.98), demonstrating the ability to exclude COVID-19 with high-confidence.

Although adversarial training only had a small effect on the overall performance of predicting COVID-19, relative to the basic model, it significantly changed the predicted probability outputs of the predictor in the adversarial model (Wilcoxon Signed Rank Test, *p* < 0.0001 for all validation cohorts).

In terms of fairness, the adversarial model achieved the best performance overall, achieving either the best equalized odds performances (for both TP and FP SDs) across all external test cohorts, except for PUH, where the false positive rate remained the same as the basic model (Table 2). Overall, equalized odds were demonstrably improved through adversarial training, with minimal trade-off (if any) in performance (AUROC increased for OUH, remained the same for BH, and decreased between 0.003–0.010 for PUH, and UHB cohorts).

**Table 2:** Equalized odds evaluation for COVID-19 prediction task (ethnicity mitigation) on prospective and external test sets, optimized to sensitivities of 0.9.

	Prospective Validation		External Validation					
	OUH (*n* = 22,857, prevalence = 8.80%)		PUH (*n* = 37,896, prevalence = 5.29%)		UHB (*n* = 10,293; prevalence = 4.27%)		BH (*n* = 1177; prevalence = 12.2%)	
	Basic	Adv	Basic	Adv	Basic	Adv	Basic	Adv
TP SD	0.0387	**0.0224**	0.0490	**0.0480**	0.0985	**0.0975**	0.0964	**0.0954**
FP SD	0.0574	**0.0539**	0.0361	0.0361	0.0510	**0.0387**	0.1039	**0.0970**

Results reported as SD of true positive and false positive rates, across all ethnicity labels. Bolded values denote best scores.

Complete performance and fairness metrics are shown in Table 2 and Fig. 1 (numerical results are shown in Supplementary Table 3).

### Debiasing Hospital

To further demonstrate the utility of our proposed method, we trained a COVID-19 prediction model that is unbiased towards the hospital a patient attended. In order to evaluate bias related to hospital location, data points from multiple sites needed to be present in the training data; thus, we combined presentations from all hospital cohorts previously described (Table 1), and used an 80:20 split to separate the data into training and test sets, respectively, stratified based on COVID-19 status and hospital cohort. This resulted in 150,304 presentations (4249 COVID-19 positive) for training and optimization, and 37,577 presentations (1052 COVID-19 positive) for testing (Table 3).

**Table 3:** Summary of number of patients, COVID-19 positive cases, and hospital case distribution for training, validation, and held-out test set cohorts used in hospital debiasing task.

	Training	Test
*n*, patients	150,304	37,577
*n*, COVID-19 positive	4249 (2.8%)	1,052 (2.8%)
Hospital:		
OUH (%)	110,906 (73.8%)	27,609 (73.5%)
UHB (%)	8224 (5.5%)	2069 (5.5%)
BH (%)	930 (0.6%)	247 (0.7%)
PUH (%)	30,244 (20.1%)	7652 (20.4%)

Clinical predictors considered (ALT: alanine aminotransferase; CRP: C-reactive protein; eGFR: estimated glomerular filtration rate).

**Fig. 2:**
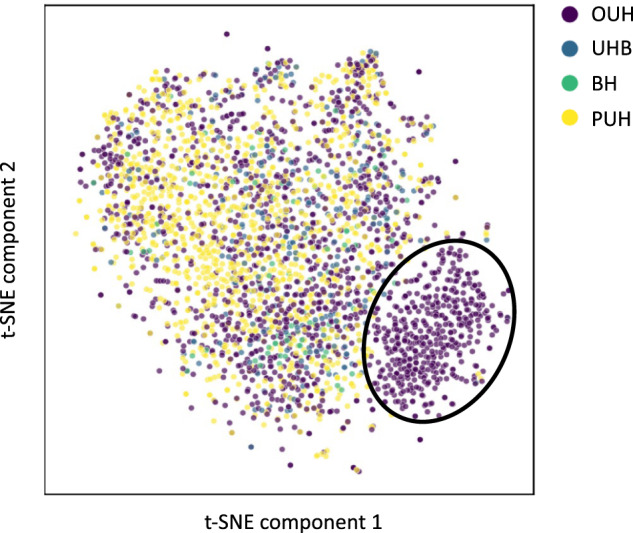
t-Stochastic Neighbor Embedding (t-SNE) visualization of data. Figure shows t-SNE representation of the training dataset used in training, including all positive COVID-19 cases across the four NHS trusts (OUH, PUH, UHB, BH).

As previously shown with ethnicity, we can see that the number of presentations available from different hospital cohorts is heavily skewed in our training dataset (Table 3). To further demonstrate the need for inter-hospital bias mitigation, we used a t-Stochastic Neighbor Embedding (t-SNE) to visualize a low-dimensional representation of all positive COVID-19 presentations in our training data (Fig. 2). From the results, we can see a distinct cluster (purple), which corresponds to a subset of the presentations from OUH. This suggests that the training data can be clustered by specific hospital locations (namely, through means of site-specific features such as annotation methods, data truncation, measuring devices, or collection/processing tools), making this feature an important and appropriate choice for bias mitigation^2,19^.

After model training, we evaluated our models on the held-out set which included patient presentations from all four hospital cohorts. Using a sensitivity configuration of 0.9, model performance for predicting COVID-19 status was consistent for both the basic and adversarial models (AUROC scores of 0.905 [0.892–0.917] and 0.902 [0.890–0.915] for basic and adversarial models, respectively). As before, these are comparable to the previous benchmarks reported, which used similar patient cohorts and features (Supplementary Table 5), demonstrating that we trained strong classifiers independent to any bias mitigation.

The optimized threshold also resulted in consistent scores for sensitivity (sensitivities of 0.876 [0.857-0.896] and 0.878 [0.859–0.898] for basic and adversarial models, respectively) and specificity (specificities of 0.760 [0.755–0.764] and 0.758 [0.753–0.762] for basic and adversarial models, respectively) across all models and cohorts. Again, both models achieved high prevalence-dependent NPV scores (>0.99), demonstrating the ability to exclude COVID-19 with high-confidence.

Relative to the basic model, adversarial training did not appear to affect the performance of predicting COVID-19 status. However, in terms of the output distribution, it significantly changed the predicted probability outputs between the two models (Wilcoxon Signed Rank Test, *p* < 0.0001).

In terms of bias mitigation, the adversarial model achieved the most fair performance, achieving the best result with respect to equalized odds (for both TP and FP SD scores). Thus, the adversarial model was able to improve equalized odds for hospital cohort, while maintaining its ability to perform the main task.

**Fig. 3:**
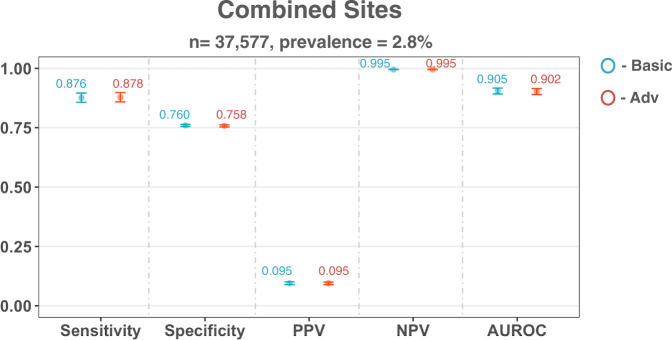
COVID-19 diagnosis performance results on dataset combining all four NHS Trusts. Panel shows performance of basic and adversarial models during validation. Adversarial models were trained to mitigate site-specific (hospital) biases. All models were optimized during training to achieve a sensitivity of 0.9. Error bars show 95% confidence intervals. Numerical results are shown in Supplementary Table 4.

Performance and fairness metrics are shown in Fig. 3 and Table 4 (numerical results can be found in Supplementary Table 4).

**Table 4.** Equalized odds evaluation for COVID-19 status prediction (hospital mitigation) on test set, threshold adjusted to sensitivities of 0.9.

	Basic	Adv
TP SD	0.0200	**0.0173**
FP SD	0.0529	**0.0480**

Results reported as SD of true positive and false positive rates, across all hospital labels. Bolded values denote best scores.

## Discussion

In this study, we demonstrated that adversarial debiasing is a powerful technique for mitigating biases in machine learning models, using a complex, real-world task - screening for COVID-19—while aiming to mitigate site-specific (hospital) and demographic (patient ethnicity) biases. We trained our framework on a large, clinically-rich COVID-19 dataset, from four independent hospital cohorts, and found that the addition of an adversary component demonstrably improved outcome fairness, without compromising performance of the task-at-hand. We know that looking at variations across different regions and ethnic groups only addresses a small subset of existing inequities in healthcare; however, the framework we outlined can be easily applied to many different tasks and features. As technological capabilities continue to grow and machine learning continues to saturate decision-making processes in healthcare, we hope that the ability to develop fair models will encourage more hospitals to adopt machine learning-based technologies, and inspire greater confidence in the utility and reliability of these tools for making critical decisions. Additionally, as we’ve demonstrated that fairness-aware ML approaches can help mitigate site-specific biases, we hope that these findings will encourage greater collaborative efforts for data curation (as ML generalizability typically relies on the availability of large amounts of data).

In general, models achieved better AUROC scores during the hospital-based bias mitigation task, compared to the ethnicity-based bias mitigation task. This was likely due to the greater amount of data available during training, emphasizing the importance of data availability, and likewise, collaborative approaches. The COVID-19 pandemic has particularly highlighted the importance of collaborations in order to rapidly respond to evolving and widespread global challenges. Thus, as machine learning datasets continue to expand, especially through collaborative efforts, there is increased potential for machine learning-based technologies. However, in parallel with model development, greater attention will need to be given to bias mitigation. We depicted this importance through using the data visualization tool, t-SNE, which demonstrated that variations between hospitals can be reflected by the data; and thus, must be considered during machine learning development and implementation. Similarly, while the outcomes from the adversarial models were less biased than the comparator models, we acknowledge that there may still be bias in the model given the population it is trained on. As our datasets are exclusively from select UK NHS Trusts, they may not be representative of other populations with distinct distributions; and thus, the final trained models and results may not be generalizable to other populations. However, because we are using neural network base models, domain adaptation (via transfer learning) can be an interesting area to explore in future studies.

We found that a neural network trained with an adversarial framework achieved consistent AUROCs when compared to a basic neural network (trained independent of any adversary component); and additionally, achieved consistent AUROCs when compared to previous XGBoost and RL benchmarks. Furthermore, as our framework is demonstrated with neural networks, this method can be generalised to image recognition problems and NLP problems, which XGBoost (and other common baselines) are typically not appropriate for. It can also be used with different model architectures, alongside transfer learning, to help improve model performance (tree-based algorithms, such as XGBoost, depend on the availability of the entire dataset, making transfer learning infeasible)^19^. Moreover, our proposed framework greatly improves on the computational cost associated with RL-based methods.

Along with being able to effectively perform the predictive task-at-hand, the outcomes of the adversarial models were less biased compared to those with no bias mitigating component. However, although bias decreased, the models did not completely satisfy equalized odds requirements (i.e., reach TP and FP SDs of zero). One factor may be that our training datasets were imbalanced with respect to the sensitive features; and since we are using neural network-based models, skewed distributions can impact classification results. This has previously been discussed^41^, as using balanced data was found to have a much stronger effect on adversarial training. Thus, future experiments would greatly benefit from balanced training data.

For COVID-19 prediction, we adjusted the decision threshold to ensure models achieved high sensitivity. This technique is especially useful when there are large imbalances in the training data (which we had in our training sets). However, as data can be biased by site-specific factors (recall the t-SNE representation), optimal thresholds can be biased on the particular dataset(s) used for derivation. Thus, the threshold used at one hospital, may not be suitable at another hospital with independent distributions. This may have contributed to the differing specificities between test sites used in the ethnicity-based bias mitigation task. The selection of an optimal decision threshold should be further investigated, as it directly affects performance and fairness metrics due to the shift of true positive/true negative rates. Particularly for clinical tasks, it is often desirable to achieve consistent sensitivity/specificity scores across different hospitals, as varying sensitivities/specificities can make it difficult for clinicians to rely on the performance characteristic of a model^2^. Future experiments can consider using site-specific thresholds which are calibrated during deployment at different sites, in order to standardise predictive performance^19^.

Similarly, the trade-off between sensitivity and specificity should be carefully considered depending on the task-at-hand. For our purposes, we optimized for sensitivity, as we focused on rapid detection; however, optimizing for specificity is also important, as a low specificity can lead to increased resource use and costs (overburdening hospitals), and well as increased anxiety/discomfort for patients. This trade-off is also important when choosing notions of fairness to evaluate, as models should be optimized to definitions of fairness that are most appropriate to each task. Moreover, as real-world datasets are imbalanced with respect to both outcome labels and sensitive feature labels, it will be beneficial to consider/develop evaluation metrics that can accommodate and reflect these different levels and types of data imbalances.

Also, bias may also still exist with respect to data missingness. Although we used imputation to “fill-in” missing values, the nature of the missing data may have conveyed important information, or reflected biases such as differences in practice or recording protocols. Thus, future studies should investigate other methods quantify and address missing data, as it may convey important information.

With respect to debiasing against ethnicity, another limitation is the ambiguity of certain categories, namely, “Unknown”, “Mixed”, and “Other.” In our experiments, we kept these categories in order to maximize the number of cases (especially COVID-19 positive cases) used in training. This may have impacted the adversary network’s ability to confidently differentiate between different ethnicities, hindering its influence on the main network. If more specific labels are available, these should be used in future models.

We also appreciate that the probability of having a disease is a useful measurement, as opposed to thresholding to a binary classification. We chose to use a binary classification rather than a probability of disease in order to match with the categorisation system implemented by NHS Trust policy; however, probability can also be used as a final output for tasks where appropriate.

Another limitation is the difficulty in understanding how social, behavioral, and genetic factors independently and collectively impact outcomes. For example, consistent genetic effects across racial groups can result in genetic variants with a common biological effect; however, that effect can also be modified by both environmental exposures and the overall admixture of the population^45^. Thus, additional evaluations into the main prediction task (and related variables) will be necessary to determine what biases exist and how to best mitigate them. For example, for certain non-clinical tasks, it is clear that ethnicity shouldn’t be a determining factor in outcome prediction (such as recidivism prediction); however, in clinical contexts, it isn’t always as clear^46^. Because we exclusively used data available from UK hospital trusts, we focused on ensuring that minority groups were not being poorly predicted due to large imbalances in the data. However, we appreciate that “ethnicity” can also incorporate notions of socioeconomic status, community, geographic region, etc., and that these factors can collectively contribute to disease prevalence among certain groups. During the early stages of a pandemic, the contribution of ethnicity (and related factors) to COVID-19 diagnosis may not be fully understood; however, as more data is collected over time, incremental adjustments should be incorporated to reflect the true contribution of such characteristics.

Finally, although issues of algorithmic fairness have been greatly discussed, there is not a consensus on a “one-fits-all” method, metric, or criterion. Thus, it can be challenging to determine and quantify the significance of existing biases, and additionally, how well they’ve been mitigated. This challenge is also compounded by the vast number of applications where issues of fairness are relevant. Thus, methods should be chosen and tailored towards each task at hand. Furthermore, upon the availability of more data, algorithms, and empirical studies, alternative methods developed will help continue to progress the field of algorithmic fairness and bias mitigation in ML.

## Methods

### Data and feature set

To train and validate our models, we used clinical data with linked, deidentified demographic information for patients across four hospital groups – Oxford University Hospitals NHS Foundation Trust (OUH), University Hospitals Birmingham NHS Trust (UHB), Bedfordshire Hospitals NHS Foundations Trust (BH), and Portsmouth Hospitals University NHS Trust (PUH). With respect to ethics approval, United Kingdom National Health Service (NHS) approval via the national oversight/regulatory body, the Health Research Authority (HRA), has been granted for development and validation of artificial intelligence models to detect Covid-19 (CURIAL; NHS HRA IRAS ID: 281832). We performed prospective validation for patients presenting to OUH, and external validation for patients admitted to BH, PUH, and UHB, emulating the real-world implementation of such a diagnostic method.

For each task, a training set was used for model development, hyperparameter selection, training, and optimization; a validation set was used for continuous validation and threshold adjustment; and after successful development and training, four held-out test sets were then used to evaluate the performance of the final models. How the data were split are detailed in following sections.

To better compare our results to previous benchmarks, we used a similar set of features to those used in^19,26,44^, which used a focused subset of routinely collected clinical features. These include blood tests (full blood counts, urea and electrolytes, liver function tests, C-reactive protein) and vital signs, excluding the coagulation panel and blood gas testing, which are not performed universally and are less informative^44^. Table 5 summarizes the final features included.

**Table 5:** Clinical predictors considered for COVID-19 diagnosis.

Category	Features
Vital Signs	Heart rate, respiratory rate, oxygen saturation, systolic blood pressure, diastolic blood pressure, temperature
Blood Tests	Haemoglobin, haematocrit, mean cell volume, white cell count, neutrophil count, lymphocyte count, monocyte count, eosinophil count, basophil count, platelets
Liver Function Tests & C-reactive protein	Albumin, alkaline phosphatase, alanine aminotransferase, bilirubin, C-reactive protein
Urea & Electrolytes	Sodium, potassium, creatinine, urea, estimated glomerular filtration rate

### Model Architecture

**Fig. 4:**
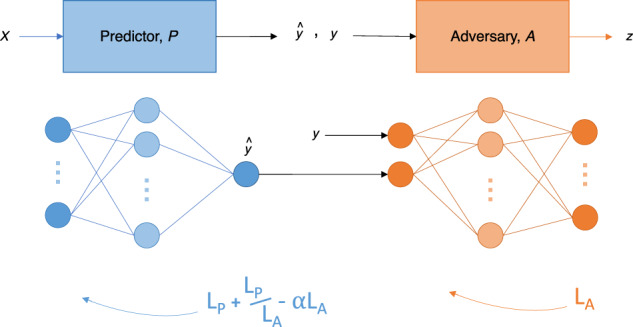
Adversarial Training Framework and Loss Functions. Figure shows the adversarial training framework used for bias mitigation. The framework consists of a predictor network (blue) and an adversary network (orange). Loss functions are also displayed alongside the respective network.

The adversarial debiasing architecture consists of two individual networks—a predictor network, *P*, and an adversary network, *A* (Fig. 4). *P* and *A* are each a multilayer perceptron (MLP) – the simplest form of a neural network. Here, *P* is trained to predict COVID-19 status (*y*), given a set of clinical features, without being biased by features, *z*. For our purposes, *z* is either hospital location or ethnicity (in machine learning literature, *z* is often referred to as the “protected” or “sensitive” feature).

Because we are training a classifier, *P*, to accurately predict *y* while satisfying an equality constraint (equality of odds), we must consider this in our training of an adversary model. As previously mentioned, equality of odds states that a classifier, *P*, is fair if ŷ and *z* are conditionally independent given *y*. Following this definition, we give the adversary model, *A*, access to both the true label, *y*, and the predicted label, ŷ; thus, limiting the information provided to *A* to those features contained in the definition. In other words, the classifier’s raw output, ŷ—the predicted probability score, and the true label, *y*, are used as the input to *A*, which tries to predict *z* (Fig. 4). Although we chose to use equality of odds, this method can be extended to other definitions as well. For example, if one wanted to train a classifier to satisfy demographic parity (which states that a classifier is fair if ŷ and *z* are independent), the adversary would be trained to predict *z*, solely given ŷ.

Our goal is to train *P* to predict *y* effectively, regardless of the demographic membership of *z*. Thus, we want *P* to be able to accurately predict *y*, and *A* to poorly predict *z*, as this suggests that *P* has been trained in such a way that debiases ŷ with respect to *z*. We use cross-entropy loss (and binary cross-entropy loss when the feature is binary), where *L*_*P*_ represents the loss for *P*, and *L*_*A*_ represents the loss for *A*.

For *P* to be good at predicting *y* while being unbiased towards *z*, *P* is typically trained to balance the trade-off between the two losses. This is achieved using the combined loss function:

1 $$L = L_P \,- \propto L_A$$

where ∝ is an adjustable hyperparameter that signifies the importance of debiasing with respect to the protected feature, *z*. This combined function encourages *P* to minimize *L*_*P*_ while maximizing *L*_*A*_. However, to ensure that *P* propagates in the correct direction at the beginning of training, we modified the combined loss function to include an attenuated correction term, such that the loss function for *P* becomes:

2 $$L = L_P + \frac{{L_P}}{{L_A}} - \propto L_A$$

Under the assumption that *L*_*A*_ starts small (*A* is able to accurately predict *z*), the correction term, $\frac{{L_P}}{{L_A}}$, ensures that *L* is large at the beginning of the training process (as $L_P + \frac{{L_P}}{{L_A}}$ is large), encouraging the adversary to increase its loss, *L*_*A*_. During training, as *L*_*P*_ becomes smaller (*P* becomes better at predicting *y*) and *L*_*A*_ becomes larger (*A* becomes unable to accurately predict *z*), $\frac{{L_P}}{{L_A}}$ → 0, converging to the original adversarial loss function, $L_P - \propto L_A$.

For *P*, the sigmoid activation function is used in the output layer (since COVID-19 prediction is a binary task); and for *A*, the softmax activation function is used instead (as the prediction of the label of *z* is a multiclass task).

We demonstrate this framework on a real-world, clinical task—COVID-19 screening using electronic health record (EHR) data from hospital EDs. Using this task, we aimed to perform bias mitigation for unwanted ethnicity-based and site-specific (hospital) biases.

### Pre-processing and hyperparameter optimization

Consistent with^19,44^, we addressed the presence of missing values by using population median imputation. As we are using a neural network base, we standardized all features in our data to have a mean of 0 and a standard deviation of 1.

To estimate which hyperparameters would perform best for each task, we performed standard five-fold cross-validation using the training data. We performed a grid search for different values of learning rate, number of hidden layer nodes in both the predictor and adversarial networks, and the dropout ratio.

Details about the software, implementation, and final hyperparameter values chosen for each model can be found in Sections A and C of the Supplementary Material.

### Threshold optimization

It is important to note that the definitions and methods introduced work with both regression and classification models, for both the main classification task and the adversary task. For our purposes, we chose to perform thresholding and use a binary classification (COVID-19 positive or negative), rather than a continuous probability score, to correspond to the green–amber–blue categorization system used by Trust policy. Here, green represented an illness with no symptoms of COVID-19, amber represented an illness with symptoms potentially characteristic of COVID-19, and blue represented a laboratory-confirmed COVID-19 infection^44^. Therefore, using classification is consistent with performing rapid triage into either a green or amber pathway.

For classification tasks, the raw output of an ML algorithm is a probability of class membership, which is then mapped to a particular class. For binary classification, values equal to or greater than 0.5 are typically mapped to one class and all other values are mapped to the other. However, this default threshold can lead to poor sensitivity, especially when the training set has a large class imbalance (as seen with our task, where there are far more COVID-19 negative cases than positive ones). Thus, we perform a grid search to adjust the threshold used for prediction, to improve detection rates at the time of testing. For our purposes, we optimized the threshold to achieve sensitivities of 0.9 to ensure clinically acceptable performance in detecting positive COVID-19 cases. This was chosen to exceed the sensitivity of lateral flow device (LFD) tests, which achieved a sensitivity of 56.9% (95% confidence interval 51.7%-62.0%) for OUH admissions between December 23, 2021 and March 6, 2021^44^. Additionally, the gold standard for COVID-19 diagnosis is by real-time PCR (RT-PCR), which has estimated sensitivities of approximately 80–90%^47,48^. Therefore, optimizing to a threshold of 0.9 ensures that the models are able to effectively detect COVID-19 positive cases (exceeding the sensitivities of current diagnostic testing methods). This threshold was also in previous studies^26,43,44^, allowing for direct comparison of results.

### Model comparators and evaluation metrics

Previously trained XGBoost^43,44^- and reinforcement learning (RL)^26^-based models provided benchmarks on which we could compare our proposed adversarial framework against (with respect to COVID-19 prediction). This allowed us to evaluate whether a model trained using an adversarial framework could effectively mitigate biases, while simultaneously ensuring that we trained a strong classifier to begin with.

We began by training a predictor network without any adversary component, which we refer to as the “basic” network (this is a standard, fully-connected neural network). This was then used as a baseline to compare the relative effects of adversarial training. Thus, we trained a set of two models—a basic (standalone predictor) model and an adversarial model—for each of the protected attributes.

For the main task of predicting the COVID-19 status of patients, we report sensitivity, specificity, positive and negative predictive values (PPV and NPV), and AUROC, alongside 95% confidence intervals (CIs) based on standard error. CIs for AUROC are calculated using Hanley and McNeil’s method^49^. Results are based on the evaluation of final, held-out test sets.

As the purpose of our framework is to train models that are unbiased towards sensitive features, *z*, we evaluate the fairness of our models using the statistical definition of equalized odds, which states that a classifier is fair if true positive rates are equal and false positive rates are equal across all possible labels of the protected variable^2–5^. As many previous works have exclusively focused on evaluating binary features, we used the standard deviation (SD) of true positive and false positive scores, enabling the assessment of multiple labels (i.e., >2). As demonstrated in^2^, we calculate true positive and false positive SD scores as follows:

3 $$\begin{array}{l}SD_{TP} = SD\left( \left\{ P\left( {{{{\hat{\mathrm Y}}}} = 1\left| {Y = 1,Z = z_i} \right.} \right),P\left( {{{{\hat{\mathrm Y}}}} = 1\left| {Y = 1,Z = z_{i + 1}} \right.} \right), \ldots ,\right.\right.\\\qquad\qquad\left.\left.P\left( {{{{\hat{\mathrm Y}}}} = 1\left| {Y = 1,Z = z_{N - 1}} \right.} \right),P\left( {{{{\hat{\mathrm Y}}}} = 1\left| {Y = 1,Z = z_N} \right.} \right) \right\} \right)\\ \quad \quad = SD\left( {\left\{ {\frac{{TP_i}}{{TP_i + FN_i}},\frac{{TP_{i + 1}}}{{TP_{i + 1} + FN_{i + 1}}}, \ldots ,\frac{{TP_{N - 1}}}{{TP_{N - 1} + FN_{N - 1}}},\frac{{TP_N}}{{TP_N + FN_N}}} \right\}} \right)\end{array}$$

4 $$\begin{array}{l}SD_{FP} = SD\left(\right.\left\{\right. P({{{\hat{\mathrm Y}}}} = 1|Y = 0,Z = z_i),P({{{\hat{\mathrm Y}}}} = 1|Y = 0,Z = z_{i + 1}),...,\\ \,\,\,\,\,\,\,\,\,\,\,\,\,\,\,\,\,\,\,\,\,\,P({{{\hat{\mathrm Y}}}} = 1|Y = 0,Z = z_{N - 1}),P({{{\hat{\mathrm Y}}}} = 1|Y = 0,Z = z_N)\left\}\right. \left)\right.\\ \quad \quad = SD\left( {\left\{ {\frac{{FP_i}}{{TP_i + FN_i}},\frac{{FP_{i + 1}}}{{TP_{i + 1} + FN_{i + 1}}},...,\frac{{FP_{N - 1}}}{{TP_{N - 1} + FN_{N - 1}}},\frac{{FP_N}}{{TP_N + FN_N}}} \right\}} \right)\end{array}$$

### Reporting summary

Further information on research design is available in the Nature Research Reporting Summary linked to this article.

## Supplementary information

Supplementary Material

REPORTING SUMMARY

## Data Availability

Data from OUH studied here are available from the Infections in Oxfordshire Research Database (https://oxfordbrc.nihr.ac.uk/research-themes/modernising-medical-microbiology-and-big-infection-diagnostics/infections-in-oxfordshire-research-database-iord/), subject to an application meeting the ethical and governance requirements of the Database. Data from UHB, PUH and BH are available on reasonable request to the respective trusts, subject to HRA requirements.

## Code availability

Code and supplementary information for this paper are available online alongside publication, and can be found at https://github.com/yangjenny/adversarial_learning_bias_mitigation.
