# Supplementary material for: An adversarial training framework for mitigating algorithmic biases in clinical machine learning
Source: NPJ Digit Med. 2023 Mar 29;6:55. doi: 10.1038/s41746-023-00805-y (PMC10050816; doi:10.1038/s41746-023-00805-y)
Supplement: Supplementary file 1 — Supplementary Material [file 41746_2023_805_MOESM1_ESM.pdf]

Supplementary Material

Algorithmic Fairness and Bias Mitigation for Clinical Machine Learning: Improvements Through Adversarial Learning

Jenny Yang, Andrew A. S. Soltan, David W. Eyre, Yang Yang, & David A. Clifton, 2023

A Software Packages and Implementation

Models were implemented using Python (v3.6.9) and PyTorch (v1.7.0). Scikit Learn (v0.24.1) was used for standardization, median imputation, and calculating performance metrics. Performance metrics were calculated using Scikit Learn and fairness metrics were manually programmed. t-SNE was implemented using Scikit Learn, with a perplexity of 40 and early exaggeration of 30. All models were run using an Intel Xeon E-2146G Processor (CPU: 6 cores, 4.50 GHz max frequency).

B Data Inclusion and Exclusion

Oxford University Hospitals NHS Foundation Trust (OUH): We included all patients attending acute and emergency care settings at OUH who received routine blood tests on arrival, considering presentations before December 1, 2019, and thus before the pandemic, as the COVID-19-negative (control) cohort. We considered presentations during the 'first wave' of the UK COVID-19 pandemic (December 1, 2019 to June 30, 2020) with PCR confirmed SARS-CoV-2 infection as the COVID-19-positive (cases) cohort. We excluded patients who opted out of electronic health record (EHR) research and those who did not receive laboratory blood tests or were younger than 18 years of age. Due to incomplete penetrance of testing during the first wave of the pandemic, and imperfect sensitivity of the PCR test, there is uncertainty in the viral status of patients presenting during the pandemic who were untested or tested negative. We therefore selected a pre-pandemic control cohort during training to ensure absence of disease in patients labelled as COVID-19-negative. Clinical features extracted for each presentation included first-performed blood tests, blood gases, vital signs measurements and PCR testing for SARS-CoV-2 (Abbott Architect [Abbott, Maidenhead, UK], TaqPath [Thermo Fisher Scientific, Massachusetts, USA] and Public Health England-designed RNA-dependent RNA polymerase assays).

Portsmouth Hospitals University NHS Foundation Trust (PUH): PUH considered all patients admitted to the Queen Alexandria Hospital, serving a population of 675,000 and offering tertiary referral services to the surrounding region, between March 1, 2020 and February 28, 2021. Confirmatory COVID-19 testing was by laboratory SARS-CoV2 RT-PCR assay, considering any positive PCR result within 48hrs of admission as a true positive.

University Hospitals Birmingham NHS Foundation Trust (UHB): UHB considered all patients admitted to The Queen Elizabeth Hospital, Birmingham, between December 01, 2019 and October 29, 2020. The Queen Elizabeth Hospital is a large tertiary referral unit within the UHB group which provides healthcare services for a population of 2.2 million across the West Midlands. Confirmatory COVID-19 testing was performed by laboratory SARS-CoV-2 RT-PCR assay.

Bedfordshire NHS Foundation Trust (BH): BH considered all patients admitted to Bedford Hospital between January 1, 2021 and March 31, 2021. BH provides healthcare services for a population of around 620,000 in Bedfordshire. Confirmatory COVID-19 testing was performed on the day of admission by point-of-care PCR based nucleic acid testing [SAMBA-II & Panther Fusion System, Diagnostics in the Real World, UK, and Hologic, USA].

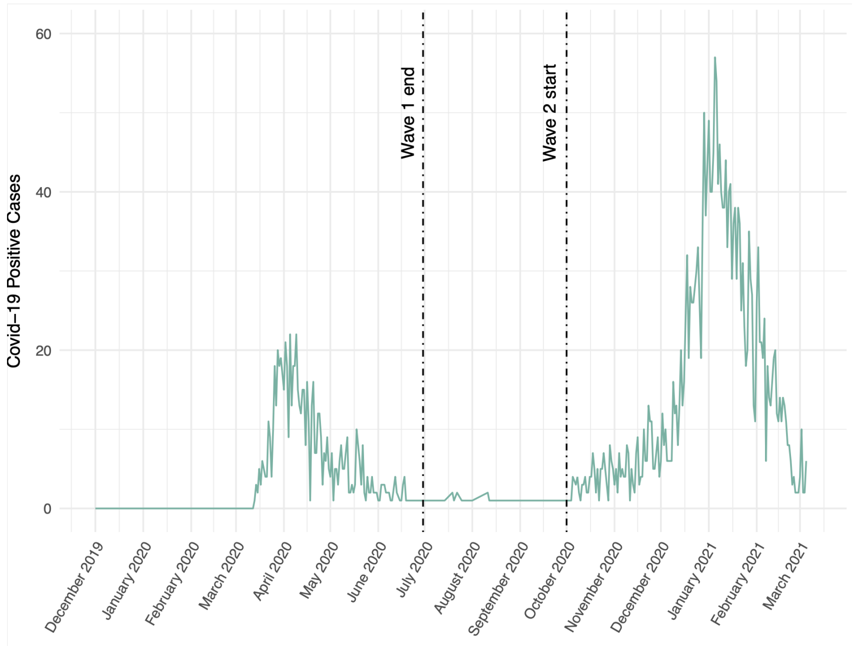

Supplementary Figure 1 : Plot of OUH positive cases, showing the first "wave" of the COVID-19 epidemic in the UK from December 1, 2019 to June 30, 2020; and the second "wave" from October 1, 2020 – March 6, 2021.

C Model Architecture

C.1 Base Neural Network Architecture

The rectified linear unit (ReLU) activation function was used for the hidden layers and the sigmoid activation function was used in the output layer for binary tasks and the softmax activation function was used in the output layer for multiclass tasks. For updating model weights, the Adaptive Moment Estimation (Adam) optimizer was used during training.

C.2 Hyperparameter Values

Supplementary Table 1 : Ethnicity-based Adversarial Training Final Hyperparameters

Learning Rate	N_p	N_adv	Dropout	Alpha	Epochs	Optimizer
1e-4	10	10	0.3	10	4000	Adam

Supplementary Table 2 : Hospital-based Adversarial Training Final Hyperparameters

Learning Rate	N_p	N_adv	Dropout	Alpha	Epochs	Optimizer
1e-4	100	10	0.3	1	4000	Adam

D Additional Results

D.1 Debiasing Ethnicity

Supplementary Table 3 : Performance of basic and adversarial models during prospective validation and external validation for ethnicity-based adversarial training. All models were optimized during training to achieve sensitivities of 0.9. Results are reported alongside 95% confidence intervals.

	Prospective Validation		External Validation					
Cohort	OUH<br>n= 22,857, prevalence = 8.80%		PUH<br>n= 37,896, prevalence = 5.29%		UHB<br>n=10,293, prevalence = 4.27%		BH<br>n=1,177, prevalence = 12.2%	
	Basic	Adv	Basic	Adv	Basic	Adv	Basic	Adv
**Sensitivity**	0.844 (0.828-0.860)	0.860 (0.845-0.875)	0.857 (0.842-0.873)	0.861 (0.846-0.876)	0.847 (0.814-0.881)	0.868 (0.836-0.900)	0.847 (0.789-0.906)	0.854 (0.797-0.912)
**Specificity**	0.710 (0.704-0.717)	0.682 (0.676-0.689)	0.672 (0.667-0.677)	0.627 (0.622-0.632)	0.716 (0.708-0.725)	0.680 (0.671-0.690)	0.822 (0.799-0.845)	0.818 (0.795-0.842)
**PPV**	0.220 (0.210-0.229)	0.207 (0.199-0.216)	0.127 (0.122-0.133)	0.114 (0.109-0.119)	0.118 (0.106-0.129)	0.108 (0.098-0.118)	0.399 (0.344-0.454)	0.396 (0.341-0.450)
**NPV**	0.979 (0.977-0.982)	0.981 (0.978-0.983)	0.988 (0.987-0.990)	0.988 (0.986-0.989)	0.991 (0.988-0.993)	0.991 (0.989-0.994)	0.975 (0.964-0.985)	0.976 (0.966-0.986)
**F1**	0.348	0.334	0.222	0.202	0.206	0.192	0.542	0.541
**AUROC**	0.866 (0.855-0.876)	0.867 (0.856-0.877)	0.867 (0.857-0.877)	0.857 (0.846-0.867)	0.867 (0.845-0.888)	0.864 (0.842-0.886)	0.894 (0.859-0.929)	0.894 (0.859-0.929)

D.2 Debiasing Hospital Location

Supplementary Table 4 : Performance of basic and adversarial models during prospective validation and external validation for hospital-based adversarial training. All models were optimized during training to achieve sensitivities of 0.9. Results are reported alongside 95% confidence intervals.

	Basic	Adv
Sensitivity (%)	0.876 (0.857-0.896)	0.878 (0.859-0.898)
Specificity (%)	0.760 (0.755-0.764)	0.758 (0.753-0.762)
PPV (%)	0.095 (0.089-0.101)	0.095 (0.089-0.100)
NPV (%)	0.995 (0.995-0.996)	0.995 (0.995-0.996)
F1	0.171	0.171
AUROC	0.905 (0.892-0.917)	0.902 (0.890-0.915)

D.3 Previous Studies

Supplementary Table 5 : Previously published COVID-19 status prediction results. using same datasets and patient cohorts. Sensitivity, specificity, and AUROC shown, alongside 95% confidence intervals, unless otherwise specified.

Test Set	Sensitivity	Specificity	AUROC
**Soltan et al., 2022.**			
Method: XGBoost + SMOTE + Threshold Adjustment (0.9)			
OUH	0.857 (SD 0.009)	0.686 (SD 0.022)	0.878 (SD 0.001)
PUH	0.841 (0.825-0.857)	0.713 (0.709 -0.718)	0.872 (0.863 -0.882)
UHB	0.788 (0.748-0.824)	0.747 (0.738 -0.755)	0.858 (0.838 -0.878)
BH	0.743 (0.666-0.807)	0.848 (0.825 0. 869)	0.881 (0.851- 0.912)
**Yang et al., 2022.**			
Method: Reinforcement Learning + Threshold Adjustment (0.9)			
OUH	0.838 (0.822-0.854)	0.707 (0.701-0.713)	0.861 (0.850-0.871)
PUH	0.828 (0.812-0.845)	0.638 (0.633-0.643)	0.831 (0.819-0.842)
UHB	0.815 (0.779-0.852)	0.717 (0.708-0.726)	0.837 (0.814-0.861)
BH	0.806 (0.741-0.870)	0.825 (0.802-0.848)	0.867 (0.829-0.906)
