# Supplementary material for: An adversarial training framework for mitigating algorithmic biases in clinical machine learning
Source: NPJ Digit Med. 2023 Mar 29;6:55. doi: 10.1038/s41746-023-00805-y (PMC10050816; doi:10.1038/s41746-023-00805-y)
Supplement: Supplementary file 2 — REPORTING SUMMARY [file 41746_2023_805_MOESM2_ESM.pdf]

nature research

Corresponding author(s): Jenny Yang

Reporting Summary

Nature Research wishes to improve the reproducibility of the work that we publish. This form provides structure for consistency and transparency in reporting. For further information on Nature Research policies, see Authors & Referees and the Editorial Policy Checklist.

Statistical parameters

When statistical analyses are reported, confirm that the following items are present in the relevant location (e.g. figure legend, table legend, main text, or Methods section).

n/a	Confirmed	
☐	☒	The exact sample size (n) for each experimental group/condition, given as a discrete number and unit of measurement
☐	☒	An indication of whether measurements were taken from distinct samples or whether the same sample was measured repeatedly
☐	☒	The statistical test(s) used AND whether they are one- or two-sided<br>*Only common tests should be described solely by name; describe more complex techniques in the Methods section.*
☒	☐	A description of all covariates tested
☐	☒	A description of any assumptions or corrections, such as tests of normality and adjustment for multiple comparisons
☐	☒	A full description of the statistics including central tendency (e.g. means) or other basic estimates (e.g. regression coefficient) AND variation (e.g. standard deviation) or associated estimates of uncertainty (e.g. confidence intervals)
☐	☒	For null hypothesis testing, the test statistic (e.g. F, t, r) with confidence intervals, effect sizes, degrees of freedom and P value noted<br>*Give P values as exact values whenever suitable.*
☒	☐	For Bayesian analysis, information on the choice of priors and Markov chain Monte Carlo settings
☐	☒	For hierarchical and complex designs, identification of the appropriate level for tests and full reporting of outcomes
☒	☐	Estimates of effect sizes (e.g. Cohen's d, Pearson's r), indicating how they were calculated
☐	☒	Clearly defined error bars<br>*State explicitly what error bars represent (e.g. SD, SE, CI)*

Our web collection on statistics for biologists may be useful.

Software and code

Policy information about availability of computer code

Data collection	No software was used here.
Data analysis	Models were implemented using Python (v3.6.9) and PyTorch (v1.7.0). Scikit Learn (v0.24.1) was used for standardization, median imputation, and calculating performance metrics. Performance metrics were calculated using Scikit Learn and fairness metrics were manually programmed. t-SNE was implemented using Scikit Learn, with a perplexity of 40 and early exaggeration of 30. All models were run using an Intel Xeon E-2146G Processor (CPU: 6 cores, 4.50 GHz max frequency). Code and supplementary information for this paper are available online alongside publication, and can be found at https://github.com/yangjenny/adversarial_learning_bias_mitigation.

For manuscripts utilizing custom algorithms or software that are central to the research but not yet described in published literature, software must be made available to editors/reviewers upon request. We strongly encourage code deposition in a community repository (e.g. GitHub). See the Nature Research guidelines for submitting code & software for further information.

Data

Policy information about availability of data

All manuscripts must include a data availability statement. This statement should provide the following information, where applicable:

- Accession codes, unique identifiers, or web links for publicly available datasets
- A list of figures that have associated raw data
- A description of any restrictions on data availability

Data from OUH studied here are available from the Infections in Oxfordshire Research Database (https://oxfordbrc.nihr.ac.uk/research-themes-overview/antimicrobial-resistance-and-modernising-microbiology/infections-in-oxfordshire-research-database-iord/), subject to an application meeting the ethical and governance requirements of the database. Data from UHB, PUH, and BH are available on reasonable request to the respective trusts, subject to NHS Health Research Authority requirements.

Field-specific reporting

Please select the best fit for your research. If you are not sure, read the appropriate sections before making your selection.

☒ Life sciences ☐ Behavioural & social sciences ☐ Ecological, evolutionary & environmental sciences

For a reference copy of the document with all sections, see nature.com/authors/policies/ReportingSummary-flat.pdf

Life sciences study design

All studies must disclose on these points even when the disclosure is negative.

Sample size	We obtained all presentations/admissions to emergency rooms across four United Kingdom National Health Service Trusts.
Data exclusions	We excluded patients who opted out of electronic health record (EHR) research and those who did not receive laboratory blood tests or were younger than 18 years of age.
Replication	We validated temporally and externally on 4 independent trusts (data availability statement added if researchers would like to access the data). Thus, we demonstrate and discuss experimental replication throughout the manuscript. We also provide the exact code used.
Randomization	Groups were based on NHS Trust. Machine learning training, validation and testing groups were randomly assigned.
Blinding	Blinding is not applicable, as this study compared results across NHS Trusts.

Reporting for specific materials, systems and methods

Materials & experimental systems

n/a	Involved in the study
☒	☐ Unique biological materials
☒	☐ Antibodies
☒	☐ Eukaryotic cell lines
☒	☐ Palaeontology
☒	☐ Animals and other organisms
☒	☐ Human research participants

Methods

n/a	Involved in the study
☒	☐ ChIP-seq
☒	☐ Flow cytometry
☒	☐ MRI-based neuroimaging
